# Establishment of a Transient and Stable Transfection System for *Babesia duncani* Using a Homologous Recombination Strategy

**DOI:** 10.3389/fcimb.2022.844498

**Published:** 2022-04-06

**Authors:** Sen Wang, Dongfang Li, Fangwei Chen, Weijun Jiang, Wanxin Luo, Guan Zhu, Junlong Zhao, Lan He

**Affiliations:** ^1^State Key Laboratory of Agricultural Microbiology, College of Veterinary Medicine, Huazhong Agricultural University, Wuhan, China; ^2^Key Laboratory of Preventive Veterinary Medicine in Hubei Province, College of Animal Science and Technology, Huazhong Agricultural University, Wuhan, China; ^3^Key Laboratory of Zoonosis Research of the Ministry of Education, the Institute of Zoonosis, and the College of Veterinary Medicine, Jilin University, Changchun, China; ^4^Key Laboratory of Animal Epidemical Disease and Infectious Zoonoses, Ministry of Agriculture, Huazhong Agricultural University, Wuhan, China

**Keywords:** *Babesia duncani*, stable transfection, gene manipulation, homologous recombination, *TPX-1*

## Abstract

Genetic modification provides an invaluable molecular tool to dissect the biology and pathogenesis of pathogens. However, no report is available about the genetic modification of *Babesia duncani*, a pathogen responsible for human babesiosis that is widespread in North America, suggesting the necessity to develop a genetic manipulation method to improve the strategies for studying and understanding the biology of protozoan pathogens. The establishment of a genetic modification method requires promoters, selectable markers, and reporter genes. Here, the double-copy gene *elongation factor-1α* (*ef-1α*) and its promoters were amplified by conventional PCR and confirmed by sequencing. We established a transient transfection system by using the *ef-1αB* promoter and the reporter gene *mCherry* and achieved stable transfection through homologous recombination to integrate the selection marker *hDHFR-eGFP* into the parasite genome. The potential of this genetic modification method was tested by knocking out the *thioredoxin peroxidase-1* (*TPX-1*) gene, and under the drug pressure of 5 nM WR99210, 96.3% of the parasites were observed to express green fluorescence protein (eGFP) by flow cytometry at day 7 post-transfection. Additionally, the clone line of the *TPX-1* knockout parasite was successfully obtained by the limiting dilution method. This study provided a transfection method for *B. duncani*, which may facilitate gene function research and vaccine development of *B. duncani*.

## Introduction

Parasites of the genus *Babesia* are prevalent apicomplexan pathogens transmitted by ticks and infect many mammals and avian species (Yabsley and Shock, 2013). Human babesiosis is mainly caused by *B. microti*, *B. divergens*, and *B. duncani* (Vannier and Krause, 2012). The infection is characterized by fever, hemolytic anemia, and even death in severe cases due to complications such as heart failure, respiratory distress, and pulmonary edema (Virji et al., 2019). Patients with immunological diseases, immunosuppressive therapy, and splenectomy are shown to have an increased risk of more severe symptoms and even death (Vannier et al., 2015). *Babesia* mainly infects people through tick bites, but an increased number of patients are reported to be infected through blood transfusions (Klevens et al., 2018; Villatoro and Karp, 2019), suggesting a huge threat of *Babesia* to human health.

In 1991, *B. duncani* was first described as strain WA1 in Washington State, USA (Quick et al., 1993), and animals such as mice, gerbils, and hamsters are susceptible to infection with *B. duncani via* intraperitoneal injection (Moro et al., 1998; Braga et al., 2006). Babesiosis caused by *B. duncani* is widespread in North America (O'Connor et al., 2018; Scott and Scott, 2018; Swei et al., 2019), and epidemiological investigations found *B. duncani* in ticks in the northeast of China (accession no. KX008042, NCBI) and South Korea (Kim et al., 2021). At the morphological level, there is no obvious difference between *B. duncani* and *B. microti* (Wozniak et al., 1996; Conrad et al., 2006). Unlike *B. microti*, *B. duncani* showed high virulence to animals (Moro et al., 1998; Kjemtrup and Conrad, 2000), causing acute death in mice and hamsters (Dao and Eberhard, 1996; Hemmer et al., 1999).

Gene editing provides a tool for biology functional study and drug target screening (Suarez et al., 2017). The development of genetic manipulation methods is necessary to improve our understanding of the basic biology of protozoan pathogens toward a better control of disease (Suarez and Noh, 2011). Previous studies described the genetic manipulation technologies in *Babesia bovis*, *Babesia gibsoni*, *Babesia ovis*, *Babesia ovata*, *Theileria annulata*, and *Theileria parva* (Adamson et al., 2001; Suarez and McElwain, 2009; Asada et al., 2012; De Goeyse et al., 2015; Hakimi et al., 2016; Liu et al., 2018)*. Babesia bovis* was the first genus to achieve stable transfection in piroplasma, with *the elongation factor 1-a B* (*Ef-1α B*) promoter being used to express the selection marker GFP-BSD, which was integrated into the *ef-1α A* region through homologous recombination (Suarez and McElwain, 2009). Later, a similar strategy was used for stable transfection in *B. gibsoni* (Liu et al., 2018), *B. ovata* (Hakimi et al., 2016), and *Babesia* sp. Xinjiang (Wang et al., 2021). For *Babesia* that infects humans, a genetic modification method was reported for *B. microti* (Jaijyan et al., 2020), but due to the lack of effective drug screening tags *in vivo*, the *B. microti* gene editing method mainly used fluorescent tags (Jaijyan et al., 2020), making it difficult to obtain *B. microti* gene-edited strains. Currently, a continuous and long-term *in-vitro* culture of *B. duncani* was established using hamster or human erythrocytes (Abraham et al., 2018; McCormack et al., 2019), while only a short-term culture can be achieved in *B. microti*. To our best knowledge, the genetic modification method has not been established in *B. duncani.* In this study, we successfully amplified the *ef-1α* regions of *B. duncani*, determined the bidirectional promoter of *ef-1α*, and established transient transfection through the *ef-1α B* promoter and the reporter gene *mCherry*, achieving the stable expression of eGFP in *B. duncani* using the WR99210/human dihydrofolate reductase gene (*hDHFR*) selection system. Moreover, we used this system to knockout the *TPX-1* gene in the *B. duncani* genome. This study provided a genetic modification method suitable for *B. duncani*, which may contribute to its research in the future.

## Materials and Methods

### Hamster Donor Blood and *Babesia duncani* Culture

Hamster blood for donor RBC was collected from hamsters into EDTA K2 (solution/RBCs = 1:9; 10% EDTA-2K), and the animals were anesthetized with isoflurane for retro-orbital venipuncture. All blood samples were centrifuged at 500×*g* for 10 min, followed by removing the plasma and buffy coat through three washes of the cells by 5 volumes of PSG, with careful removal of the supernatant and buffy coat at each wash. Next, the washed RBC was stored at 4°C for a maximum of 2 weeks in an equal volume of PSG plus extra glucose (20 g glucose/L) with a final concentration of 200 μg/ml streptomycin and 200 U/ml penicillin.

*Babesia duncani* strain WA1 (ATCC PRA-302™) was obtained from the ATCC and maintained in our laboratory (State Key Laboratory of Agricultural Microbiology, College of Veterinary Medicine, Huazhong Agricultural University, China). *Babesia duncani* was cultured *in vitro* (Abraham et al., 2018; McCormack et al., 2019) in the presence of HL-1 supplemented with 1 mg/ml AlbuMAX I (Gibco Life Technologies, Shanghai, China), 200 μM L-glutamine (Sigma, Shanghai, China), 2% antibiotic/antimycotic 100× (Corning, Shanghai, China), and 20% FBS at 37°C in a microaerophilous stationary phase (5% CO_2_, 2% O_2_, 93% N_2_).

### Cloning and Sequencing of the *ef-1a* Locus of the *Babesia duncani* WA1 Strain

According to previous reports, a putative glutamyl tRNA synthase gene was located next to the *ef-1α B* gene, and a gene homologous to the putative ribonucleotide reductase R2 subunit was located downstream of the *ef-1α A* gene (Suarez et al., 2006). To confirm that *ef-1α* is a double-copy gene, we designed primers on two adjacent genes (**Table 1**). The primer of *ef-locus-1* is located in the *ef-1α* gene, and the primers of *ef-locus-2* and *ef-locus-3* are located in the *ribonucleotide reductase R2 subunit* gene and the *glutamyl tRNA synthase* gene upstream of *ef-1a A* and downstream of *ef-1a B*, respectively. Using these three primers for PCR amplification and sequencing, we confirmed that *B. duncani* has two copies of *ef-1α*, but failed to obtain the sequence between the two copies. To obtain the sequence between the two copies, four more primers (*ef-locus-4*, *ef-locus-5*, *ef-locus-6*, and *ef-locus-7*) with a higher annealing temperature of 72°C were designed at the *ef-1α* location, and using these four primers, the intergenic (IG) region was successfully amplified and sequenced. For the analysis of the *ef-1α* locus of the *B. duncani* WA1 strain, we obtained two overlapping fragments by PCR amplification (*ef-locus-8*, *ef-locus-9*) of *B. duncani* genomic DNA extracted from *in-vitro*-cultured parasites using a genomic DNA purification kit (Tiangen, Beijing, China).

**Table 1:** Primers used in this study.

Primer	Sequence	Target
ef-locus-1	GCTACTTGAAGAAGGTTGG	Cloning and sequencing of the *ef-1a* locus
ef-locus-2	CATCAATAATTTGCTGCTGCC	
ef-locus-3	CATCTGCATTCAGTTTATTCC	
ef-locus-4	GGCTCCATCATATCCAAGGCCTCCACCAAAGTCTTACCC	
ef-locus-5	GGCACCTTCTCAATGTTGTAGCCAACCTTCTTCAAGTAGCCGC	
ef-locus-6	CCACCAGCTTCAGCTGGTACGACAAGCATAGCCACATCAGCCTG	
ef-locus-7	CCGGTAGTTGTCGACTTTCCACTGTCGACGTGACCGATGACGACC	
ef-locus-8	CGGATTTCAAAATTATTTAATAGTGG	
ef-locus-9	GGTATTAATTCTAATTGTCTCCACG	
pBS-F	AAGCTTATCGATACCGTCGA	*pBS-MER*
pBS-R	CGGGGGATCCACTAGTTCTAGAGC	
pbs-ef-B-F	GTCGACGGTATCGATAAGCTTTTTCTTTACTCAAGAAAATG	
pbs-ef-B-R	TCCTCGCCCTTGCTCACCATTTTGTTCAATTGAAACTATA	
EGFP/mCherry-F	ATGGTGAGCAAGGGCGAGGA	
EGFP/mCherry-R	TGGACGAGCTGTACAAGTAA	
pbs-rap-f	TGGACGAGCTGTACAAGTAATCAAATAAAACTAATAATAA	
pbs-rap-r	CTAGTGGATCCCCCGGGATATCCAATTGTGGATAATCACA	
ef-hDHFR-r	gcagtttagcgaaccaaccatTTTGTTCAATTGAAACTATATC	*pBS-EHEG*
hDHFR-F	atggttggttcgctaaactgc	
hDHFR-R	atcattcttctcatatacttc	
hDHFR-EGFP-F	gaagtatatgagaagaatgatATGGTGAGCAAGGGCGAGGAGC	
EGFP-ef-R	GTTTCTATTGTAATTTCAGTTTACTTGTACAGCTCGTCCAT	
efB-3H-F	ACTGAAATTACAATAGAAAC	
efB-3H-R	TAGAACTAGTGGATCCCCCGGATTAGCCTATTTGCACATGC	
tpx-locus-f	TTCGTGAGAGGATGGCTCAA	*tpx-1* sequencing
tpx-locus-r	TGAATGTTCAATGGCCCCTA	
tpx-5H-F	TCGACGGTATCGATAAGCTTATGATACCAAGATATTTGTA	*pBS-DHFR-EGFP-TPX-1 KO*
tpx-3H-R	GAAATTTTCGTGCCACTGCTAAGTTTCTAATGACTAAAAATTAC	
tpx-3H-F	TGGACGAGCTGTACAAGTAAAGTTTAACATTTTAATGTGG	
tpx-3H-R	GGCTGCAGGAATTCGATATCTAGGGATTGTGAAAAGAGAA	
pcr-1-hDHFR	cagtttagcgaaccaaccat	
Pcr-2-EGFP	CGGGATCACTCTCGGCATGG	
pcr-1-ef	GGTATTAATTCTAATTGTCTCCACG	
pcr-2-ef	CATCAATAATTTGCTGCTGCC	
pcr-3-ef	GCTACTTGAAGAAGGTTGG	With pcr-2-ef
pcr-1-tpx	GCAGATGTGATTGCAAACTC	
pcr-2-tpx	GGTCTCCATGGACCGGTAG	
pcr-3-tpx	GCAGTTATGCCAGACAATTC	With pcr-2-*TPX-1*

### Cloning and Sequencing of the *TPX-1* Locus of the *Babesia duncani* WA1 Strain

Disrupting the *TPX-1* gene requires the accurate genomic location of the *TPX-1* gene, so two primers (*tpx-locus-f* and *tpx-locus-r*) were designed to amplify the *TPX-1* gene. We obtained one fragment by PCR amplification of *B. duncani* genomic DNA, and the PCR product was sequenced directly.

### WR99210 Efficacy on *Babesia duncani In-Vitro* Culture

*Babesia duncani* was cultured in 96-well plates with various concentrations of WR99210 (20, 10, 5, 2.5, 1.25, 0.6125, 0.306, 0.15, 0.075, and 0 nM). Parasite growth was determined by the SYBR Green assay as previously reported (Abraham et al., 2018). The normalized luciferase activities were plotted using GraphPad Prism 6.

### Plasmid Constructs

**Figure 1:**
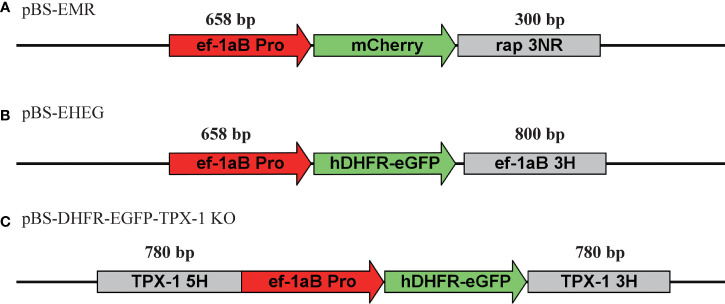
Schematic diagram of plasmid constructs for transient and stable transfection. **(A)** pBS-EMR was designed for the establishment of an *in-vitro* transient transfection system of *Babesia duncani.* **(B)** pBS-EHEG was designed for *B. duncani* stably expressing hDHFR-eGFP. **(C)** pBS-DHFR-EGFP-TPX-1 KO was designed for disruption of the *B. duncani TPX-1* gene.

The plasmid schematic diagrams used in this study are shown in **Figure 1**. For *pBS*-*EMR*, the *ef-1α B* promoter and *rap-1* terminator were amplified from the *B. duncani* genome, the *mCherry* fragment was amplified from the plasmid of *pBS-PAC-mCherry*, and these fragments were cloned separately into the *pBluescript* (*pBS*) backbone plasmid using the ClonExpress MultiS One Step Cloning Kit (Vazyme, China). The plasmid of *pBS-EHEG* was modified on the basis of plasmid *pBS-EMR*, replacing the *mCherry* gene and 3′UTR of *rap-1* with the *hDHFR-eGFP* and 3′UTR of *ef-1αB* by the ClonExpress MultiS One Step Cloning Kit (Vazyme, China). The plasmid of *pBS-DHFR-EGFP-TPX-1 KO* was cloned using the same method. Briefly, on the basis of the plasmid *pBS-EHEG*, the 5′UTR of the *TPX-1* gene was inserted into the upstream region of the *ef-1α B* promoter and the 3′UTR of *ef-1αB* was replaced with the 3′UTR of *TPX-1*. All plasmids were sequenced to confirm the accuracy of the sequence. All primers used in this study are shown in **Table 1**.

### Transfection of Parasites and Drug Selection

To prepare *B. duncan*i-infected RBCs (iRBCs) for transfection, cell pellets were washed twice with PBS and once with cytomix buffer (120 mM KCl, 0.15 mM CaCl_2_, 10 mM K_2_HPO_4_, 10 mM KH_2_PO_4_, 25 mM HEPES, pH 7.6, 2 mM EGTA, and 5 mM MgCl_2_). Electroporation was performed in BTX using 0.2 cm cuvettes containing filter-sterilized cytomix buffer at a final volume of 200 l, and parameters for transfection were 1,200 V, 25 μF, and 2 times, with 50 μg plasmid in 100 μl iRBC and 100 μl cytomix buffer.

After transfection, the mixtures were transferred into 24-well culture plates containing 5% fresh RBCs and 20% FBS, followed by incubation for 24 h and the addition of 5 nM WR99210 (MCE, Shanghai, China). To obtain a clonal strain, 1 μl iRBC was collected at 10% parasitemia and diluted to 6 infected RBCs/ml with completed medium containing 5% of fresh RBCs. To make the clonal line, 0.6 iRBC was added into each well with a total volume of 100 μl in a 96-well culture plate, replacing 70 μl culture medium every 3 days until the parasitemia reached 1%. After 12 days of culture, parasites could usually be observed, and the culture was transferred to a 48-well plate for further analysis.

### Analysis of Recombinant Parasites by PCR

To select the recombinant monoclones, blood samples of recombinant parasites were collected in the 96-well plate, and RBCs were lysed using 0.1% saponin in phosphate buffer saline (PBS), followed by boiling the sample in boiling water for 10 min and using it directly for PCR. Parasite genomic DNAs were isolated with DNeasy blood kits and used for PCR amplification.

Three pairs of primer (**Table 1**) (*pcr-1-hDHFR*; *pcr-2-EGFP*; *pcr-1-ef*; *pcr-2-ef*; *pcr-3-ef*) were designed to confirm the integration of *hDHFR-eGFP* into the *ef-1αB* locus. The position of the primer and the size of the product are shown in **Figure 5**. The same method (three pairs of primer: *pcr-1-hDHFR*; *pcr-2-EGFP*; *pcr-1-tpx*; *pcr-2-tpx*; *pcr-3-tpx*) was used to identify the *TPX-1* KO strain. All primers are shown in **Table 1**.

### Fluorescence Analysis of Recombinant Parasites

For live-cell imaging, parasite-infected blood was washed twice with PBS, followed by staining the cells with 1 μg/ml Hoechst 33342 (Sigma, Shanghai, China) in PBS. All images were captured and processed using identical settings in the OLYMPUS FRAME_BX63 scanning confocal microscope with a ×100 numerical-aperture (NA) oil objective.

To quantify the proportion of GFP-positive parasites, *TPX-1* KO parasites were washed with PBS and analyzed with a flow cytometer. The cell nuclei were first stained with 2 μg/ml Hoechst 33342 (Sigma, Shanghai, China) and 2 μg/ml PI (propidium iodide) in PBS, with PI being used to count the dead cells. After a single wash, 100,000 cells were counted on the CytoFLEX LX flow cytometer, followed by selecting the cell populations without red fluorescent signal for data analysis using CytExpert 2.4, with gating for nuclear stain Hoechst 33342 and green fluorescence.

### Western Blotting

To collect parasites, RBCs were lysed using 0.1% saponin in PBS to wash and remove the hemoglobin. Total proteins extracted from parasite pellets were separated on 12.5% SDS-polyacrylamide gels and transferred to polyvinylidene difluoride (PVDF) membranes (GE, Shanghai, China), followed by incubation with blocking buffer (TBST with 5% skimmed milk) at room temperature for 1 h and then at 4°C overnight with anti-GFP (rabbit; 1:5,000; Proteintech, Shanghai, China). Next, the PVDF membranes were incubated with horseradish peroxidase-conjugated goat anti-rabbit for 2 h at room temperature, followed by three washes with blocking buffer to enhance chemiluminescence (Sahi et al., 2009) detection, cutting the membrane between 30 and 45 kDa, and incubation with anti-GAPDH (rabbit; 1:2,000; Proteintech, Shanghai, China). The antibody of GAPDH was used as the internal control.

## Results

### Identification of the *ef-1α* Locus

**Figure 2:**
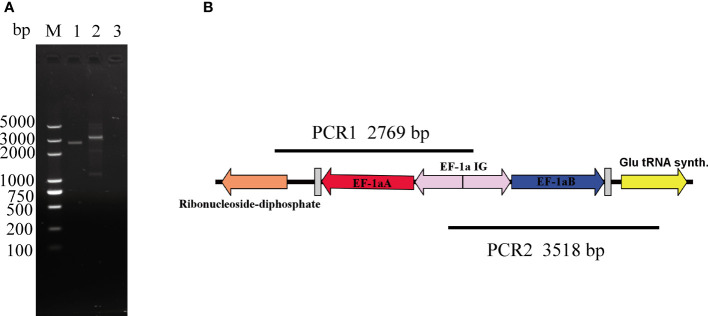
Organization of the *ef-1α* locus. **(A)** DNA agarose gel image of two overlapping fragments, with line 1 for the product of PCR1, line 2 for the product of PCR2, and line 3 for the negative control. **(B)** Location information of *ef-1α*. Glutamyl tRNA synthase gene is located next to the *ef-1α B* gene and ribonucleotide reductase R2 subunit is located downstream of the *ef-1αA* gene.

The promoter of the *ef-1α* gene is most commonly used in various apicomplexan protozoa (Adamson et al., 2001; Suarez and McElwain, 2009; Asada et al., 2012; De Goeyse et al., 2015; Hakimi et al., 2016; Liu et al., 2018), and the *ef-1α* gene has two copies in the genome, providing a suitable site for the stable transfection of the gene. However, due to the lack of genomic data, the *ef-1* gene promoter sequence remains unknown. By comparing multiple sequences of *Babesia* and designing specific PCR primers, we successfully obtained the locus of the *ef-1α* gene. For the analysis of the *ef-1α* locus of the *B. duncani* WA1 strain, two overlapping fragments were obtained by PCR amplification of *B. duncani* genomic DNA (**Figure 2**). Similar to *B. bovis* (Suarez et al., 2006), the *ef-1a* locus in *B. duncani* contains two identical *ef-1α* genes (denoted as *ef-1αA* and *ef-1αB* in **Figure 2**). Both *ef-1α* ORFs are 1,347 bp, arranged in a head-to-head orientation and separated by a 1,302-bp IG region. The sequence information of *ef-1α* obtained in this study was submitted to GenBank with the accession number OL804102.

### Establishment of an *In-Vitro* Transient Transfection Method of *Babesia duncani* Parasite

**Figure 3:**
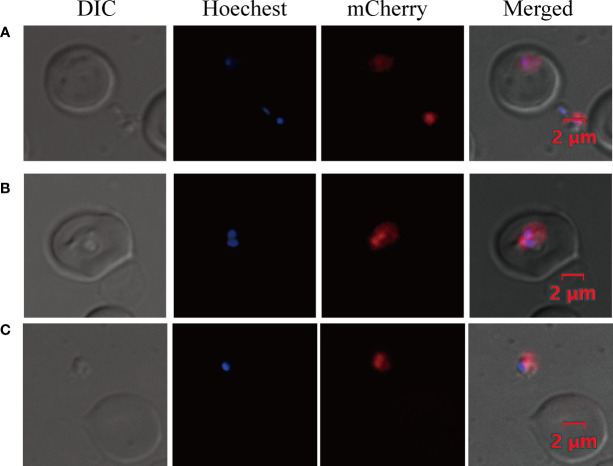
Transient transfection of *B. duncani*. *Babesia duncani* was electroporated with plasmid pBS-EMR and detected by live fluorescence microscopy at 24 h post-transfection. Red fluorescence corresponds to the transfected parasite expressing *mCherry*, Hoechst staining represents the nucleus of the parasite, and the DIC image shows a parasitized RBC **(A–C)**. The merged image represents the overlap of all images. Scale bar = 2 μm.

The success of an efficient transfection method requires effective promoters and a suitable strategy for DNA transfection (Suarez and McElwain, 2010). To establish the *B. duncani* transfection method, a plasmid (*pBS-EMR*) expressing *mCherry* was constructed, using 658-bp 5′UTR of *ef-1α B* as the promoter and 300-bp 3′UTR of *rap-1* as the terminator. Based on the *B. bovis* transfection method (Suarez and McElwain, 2008), we have made some modifications to achieve higher transfection efficiency. Electroporation was performed in BTX with 0.2 cm cuvettes containing filter-sterilized cytomix buffer at a final volume of 200 μl, using the parameters of 1,200 V, 25 μF, and 2 times for transfection, with 50 μg plasmid in 100 μl iRBC and 100 μl cytomix buffer. At 24 h post-transfection, the parasites expressing *mCherry* were observed by live fluorescence microscopy (**Figure 3**), proving the feasibility of this electroporation method.

### The Inhibitory Effect of WR99210 on *Babesia duncani In-Vitro* Culture

**Figure 4:**
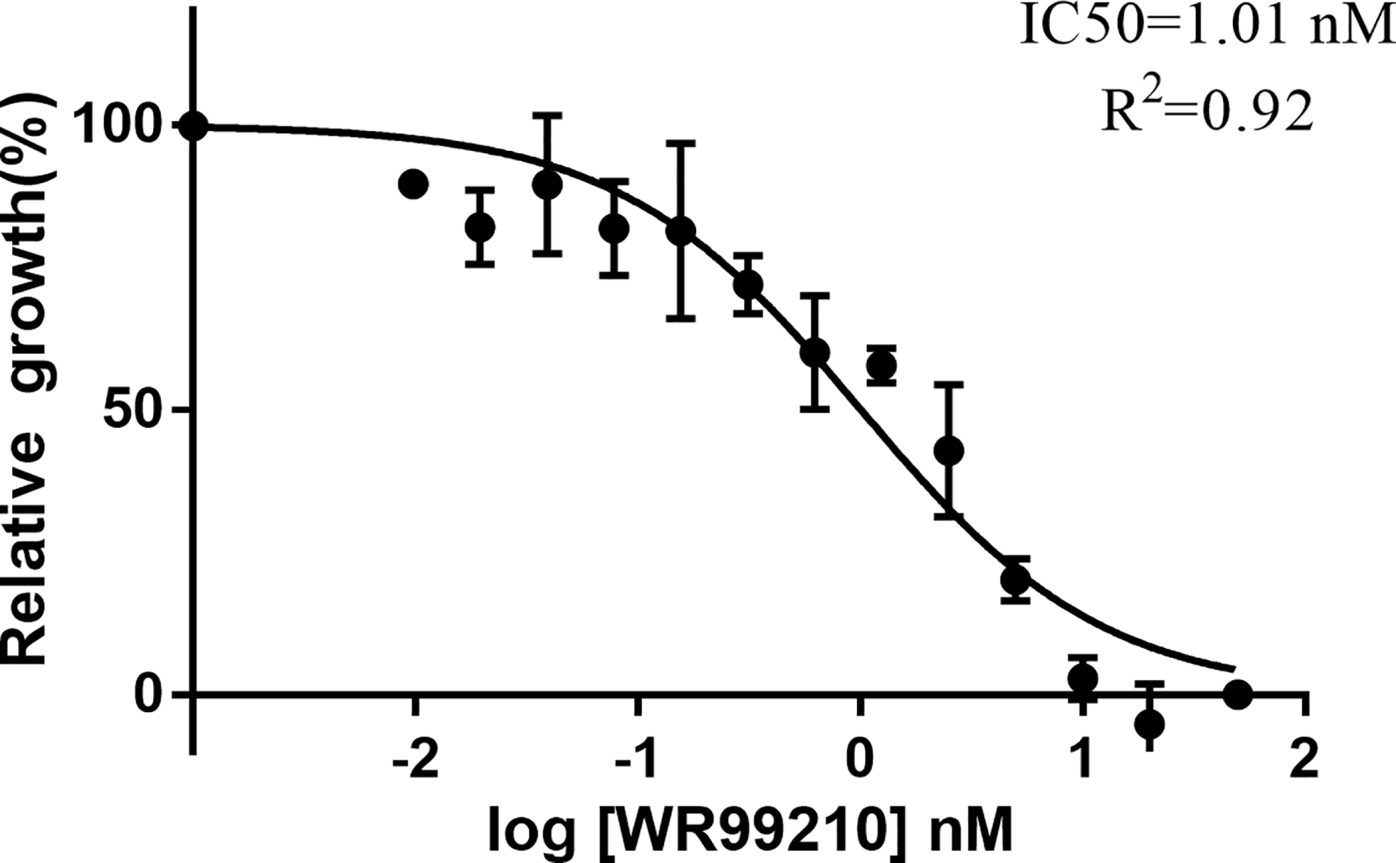
Growth inhibition of *B. duncani* by WR99210. Evaluation of the susceptibility of *B duncani in vitro* to WR99210 at concentrations of 20 to 0.075 nM. All data are presented as means  ± SD of triplicate cultures (*n* = 3).

To examine the inhibition of WR99210 on *B. duncani*, the parasites were cultured with different concentrations of WR99210 (0 to 20 nM). The experiment was performed in triplicate wells, and parasitemia was calculated on day 3 after adding the drug into the culture. The IC50 of WR99210 was 1.01 nM, and 5 nM WR99210 could inhibit 80% of the growth of *B. duncani* (**Figure 4**), indicating that 5 nM WR99210 could be used to select genetically modified parasites.

### Establishment of *Babesia duncani* With Stable Expression of *hDHFR-eGFP*

**Figure 5:**
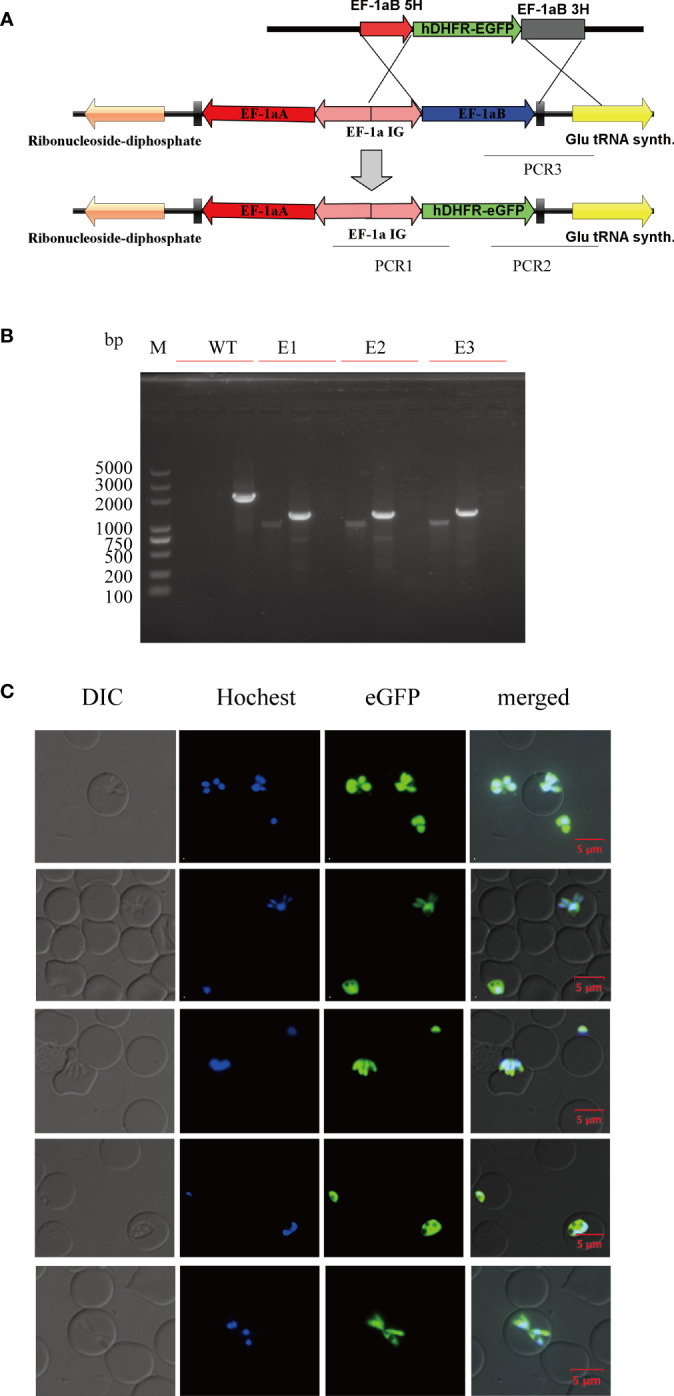
Establishment of the *B. duncani* line with stable expression of hDHFR-eGFP. **(A)** Plasmid construct for stable eGFP expression (pBS-EHEG): the recombination sites for integration of *pBS-EHEG* into the ef-1α locus through homologous double cross-over recombination, with the sites being shown for PCR1, PCR2, and PCR3. **(B)** PCR confirmation of the integration of *pBS-EHEG* into the ef-1α locus. Monoclonal strains (E1, E2, and E3) were identified by PCR1, PCR2, and PCR3, with the WT strain used as the control. **(C)** Green fluorescence corresponds to the transfected parasite expressing eGFP, Hoechst staining represents the nucleus of the parasite, and the DIC image shows a parasitized RBC. The merged image represents the overlap of all images. Scale bar = 5 μm.

At 12 days post-selection by 5 nM WR99210, eGFP-expressing parasites appeared in cultures transfected with linearized plasmids (**Figure 5A**). The integration of *hDHFR-eGFP* into the *ef-1α B* locus was initially evaluated by PCR. After confirming the integration event, the transfected *B. duncani* clone line was obtained by limiting dilution. PCR1, PCR2, and PCR3 primer pairs could successfully amplify 1,031, 1,273, and 2,066 bp fragments in the clone line, respectively, but not in the wild type (WT) of *B. duncani* (**Figure 5B**). *hDHFR-eGFP* expression was detected by live fluorescence microscopy (**Figure 5C**), and fluorescence could be observed in the cytoplasm of different forms of parasites. Additionally, the growth curves showed no significant difference between the WT and eGFP parasites (**Figure S1A**). These results suggested that eGFP can be stably expressed in *B. duncani*, with no side effect on the growth of the parasites.

### Targeted Disruption of the *Babesia duncani TPX-1* Gene

**Figure 6:**
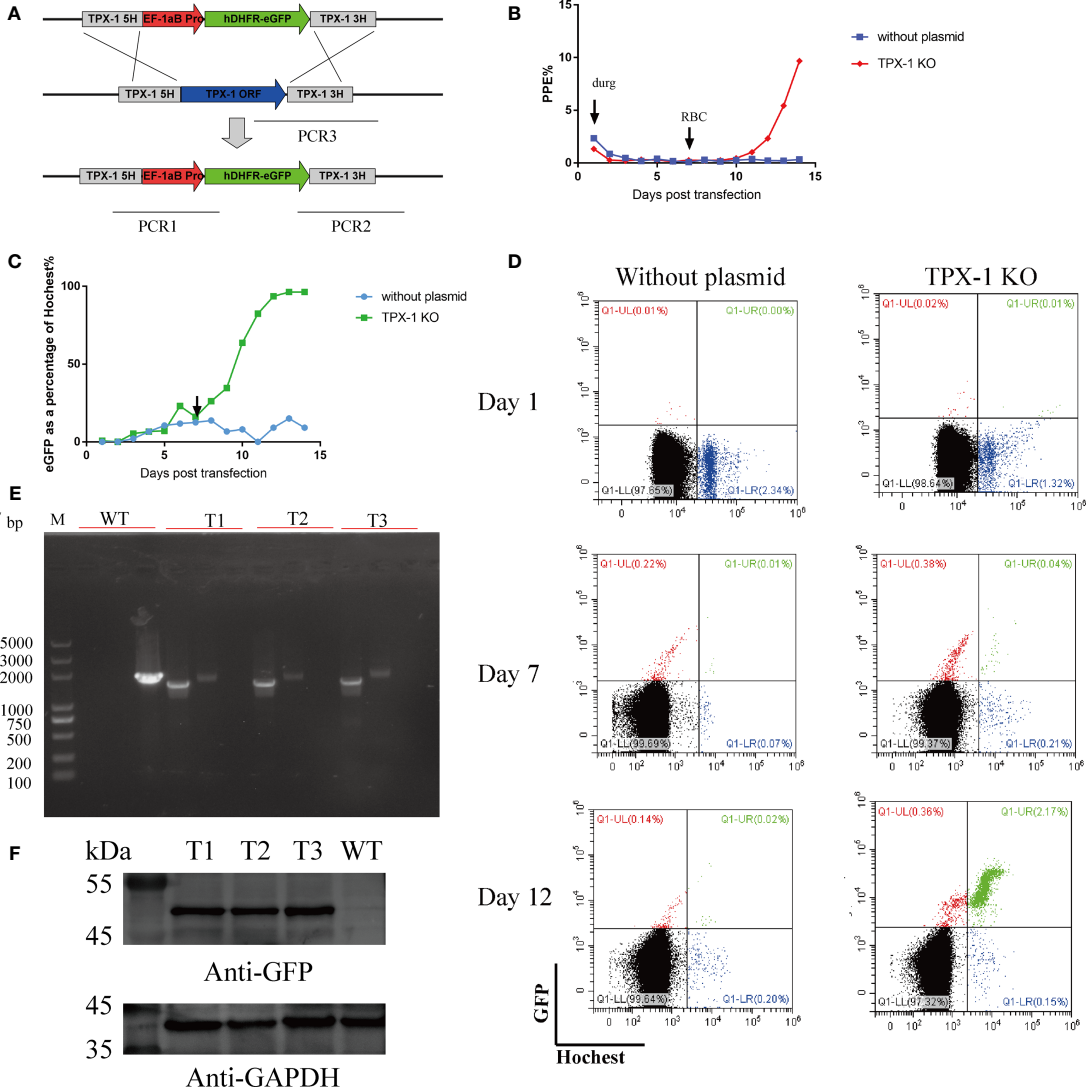
Targeted disruption of the *B. duncani TPX-1* gene. **(A)** Plasmid construct for disruption of the *B. duncani TPX-1* gene (*pBS-DHFR-EGFP-TPX-1 KO*): the recombination sites for integration of *pBS-DHFR-EGFP-TPX-1 KO* into the TPX-1 locus through homologous double cross-over recombination. **(B–D)** The results of flow cytometry. PPE was estimated by Hoechst staining, with 5 nM WR99210 being added on day 1 and RBC added on day 7. The proportion of parasites expressing green fluorescent protein was recorded, and flow cytometry results on days 1, 7, and 12 were displayed, with the abscissa for Hoechst staining and the ordinate for green fluorescence. Approximately 0.75% of parasites could express fluorescent protein at 24 h post-transfection, and parasites with stable eGFP expression could be observed at day 6 post-drug screening. **(E)** PCR confirmation of the disruption of the *B. duncani TPX-1* gene. Monoclonal strains T1, T2, and T3 were identified by PCR1, PCR2, and PCR3, with the WT strain used as the control. **(F)** hDHFR-eGFP expression detected by immunoblotting with anti-GFP antibody. The expression of hDHFR-eGFP could be detected in all monoclonal strains, but not in the WT strain.

The gene manipulation ability of this method for *B. duncani* was tested by disrupting the *TPX-1* gene through homologous recombination established in this study. The 780-bp 5′UTR and 780-bp 3′UTR of the *TPX-1* gene were used as the homology arms, and *hDHFR* was used as the drug selection marker (**Figure 6A**). After transfecting the parasites with circular plasmids, the daily PPE (**Figure 6B**) and the proportion of eGFP expression were calculated by flow cytometry (**Figures 6C, D**). Approximately 0.75% of parasites could express the fluorescent protein at 24 h post-transfection. After 13 days of drug screening, 96.3% of parasites could stably express eGFP and show green fluorescence (**Figures 6C, D**). Resistant parasites were observed by Giemsa staining on day 7, and parasites with green fluorescence could be easily observed by flow cytometry (**Figure 6D**). To obtain the clonal line, transfected *B. duncani* was subjected to limiting dilution. After 12 days of culture, 11 clone lines were obtained, which were identified by PCR, indicating that the *TPX-1* gene was successfully knocked out (with only 3 clone lines shown in the results) (**Figure 6E**). The PCR1, PCR2, and PCR3 primer pairs could successfully amplify 1.6, 2.0, and 2.1 kbp DNA fragments in the KO clone line, respectively, but not in *B. duncani* WT (**Figure 6E**). Western blot results also confirmed the expression of the eGFP protein, in contrast to no signal for WT (**Figure 6F**). Meanwhile, no obvious difference was observed in the growth curves between the WT and *TPX-1* KO parasites (**Figure S1B**).

## Discussion

Gene editing is an important method to study the cell biology of parasites and gene functions (Suarez et al., 2017). The application of a transfection system can also facilitate a better understanding of the mechanisms underlying drug resistance and host–parasite interactions, thus providing novel information for vaccine development and drug target discovery (Alzan et al., 2019; Gallego-Lopez et al., 2019). The first stable transfection system in the *Babesia* genus was reported in *B. bovis* in 2009 (Suarez and McElwain, 2009), followed by *B. gibsoni* and *B. ovata.* However, there is no report about the genetic manipulation of *B. duncani.* Here, we described a stable transfection system for *B. duncani*, an important zoonotic parasite that infects humans and rodents (Vannier and Krause, 2012). For human *Babesia*, the main pathogen is *B. microti*, which cannot be cultured for a long time *in vitro* due to a lack of effective drug screening labels *in vivo*, limiting the development of its gene editing technology. The continuous and long-term *in-vitro* culture of *B. duncani* had been established by using hamster or human erythrocytes (Abraham et al., 2018; McCormack et al., 2019), enabling us to study *Babesia* more conveniently and develop new drugs and vaccines.

The success of an efficient transfection method requires effective promoters and a suitable strategy for DNA transfection (Suarez and McElwain, 2010). The promoter of *ef-1α* is efficient, leading to its wide use in the transfection of a variety of organisms, including *B. bovis*, *B. gibsoni*, *B. ovata* (Suarez and McElwain, 2009; Hakimi et al., 2016; Liu et al., 2018), and *Babesia* sp. Xinjiang (Wang et al., 2021). Here, we successfully amplified the *ef-1α* region, and sequencing analysis proved that *ef-1* is a double-copy gene, with a head-to-head pattern between the two copies and separated by a 1.3k region of IG. Bioinformatics analysis suggested that the 667-bp upstream of *ef-1α A* and the 658-bp upstream of *ef-1α B* are the promoters of each gene. The latter was used as the promoter to establish the transfection method, leading to the successful expression of the fluorescent protein and drug screening tag in the transient and stable transfection system.

Most previously reported transfection systems for *Babesia* focused on bovine *Babesia* species using the Gene Pulser Xcell™ electroporation system (Bio-Rad, VA, USA) and Amaxa Nucleofector™ 2b device (Lonza) (Suarez and McElwain, 2008), paying little attention to the transfection strategy of *B. duncani*. In this study, electroporation was performed in BTX, using the parameters of 1,200 V, 25 μF, and 2 times. This strategy can be used for stable transfection, but the initial transfection efficiency was detected to be only between 10^−3^ and 10^−2^ by flow cytometry analysis, suggesting that more research efforts should be made to improve the transfection efficiency to obtain genetically modified parasites more quickly.

A drug selection marker is essential to the establishment of a stable transfection system. In this study, the sensitivity of *B. duncani* to WR99210 was evaluated. WR99210 was previously reported for drug screening in *Plasmodium* (de Koning-Ward et al., 2000), *B. bovis* (Asada et al., 2012), and *B. gibsoni* (Liu et al., 2018). Our results showed that *B. duncani* was extremely sensitive to WR99210 with an IC50 of 1.2 nM, close to the IC50 value of *B. bovis* (1 nM) (Asada et al., 2015) and *B. gibsoni* (1.1 nM) (Liu et al., 2018) and almost twice the value of *B. ovata* (0.56 nM) (Hakimi et al., 2016). The sensitivity of *B. duncani* to blasticidin S deaminase (BSD) was also evaluated, and *B. duncani* was resistant to BSD, whose growth could not be inhibited even at 100 μg/ml BSD. This may be related to the transport carrier of *B. duncani* (Mira-Martínez et al., 2013).

In this study, we successfully replaced *ef-1αB* and *TPX-1* with linearized plasmids and circular plasmids. The disruption of these two genes did not affect the growth of *B. duncani* at the blood stage, which was consistent with the previous report of *B. bovis* (Asada et al., 2015). These results indicate that genetic manipulation in this study did not affect the growth of the parasite *in vitro*. *Babesia duncani* could infect humans and perform better as an animal model than other *Babesia*, so *B. duncani* is more suitable for studying some virulence or immune regulation genes. Meanwhile, there are still some defects in this system, including low knockout efficiency relative to CRISPR/Cas9-based genome editing strategies, but this *B. duncani* transfection system provides a useful tool for determining gene function and discovering critical gene families related to invasion, egress, immune evasion, and even virulence factors. On this basis, a more convenient, facile, and highly effective technique can be expected to be developed in the near future.

## Conclusion

In this study, we established a genetic modification tool for *B. duncani* and successfully integrated exogenous genes into the *B. duncani* genome. This *B. duncani* genetic modification tool may facilitate the determination of gene functions, discovery of novel drug targets, establishment of infection models, and evaluation of the interactions between the parasite and the host.

## Data Availability Statement

The datasets generated for this study can be found in the NCBI GenBank under the accession number OL804102. The original contributions presented in the study are included in the article/**Supplementary Material**, further inquiries can be directed to the corresponding author.

## Ethics Statement

This study was approved by the Scientific Ethics Committee of Huazhong Agricultural University (permit number: HZAUMO-2017-040). All mice were handled in accordance with the Animal Ethics Procedures and Guidelines of the People’s Republic of China. Written informed consent was obtained from the owners for the participation of their animals in this study.

## Author Contributions

SW, LH, and JZ designed the study and wrote the draft of the manuscript. DL, FC, WJ, and WL performed the experiments and analyzed the results. All authors have read and approved the final manuscript.

## Funding

This work was supported by the National Natural Science Foundation of China (Grant Nos. 31930108 and 31772729) and the Fundamental Research Funds for the Central Universities in China (Project2662020DKPY016).

## Conflict of Interest

The authors declare that this study was conducted in the absence of any commercial or financial relationships that could be construed as a potential conflict of interest.

## Publisher’s Note

All claims expressed in this article are solely those of the authors and do not necessarily represent those of their affiliated organizations, or those of the publisher, the editors and the reviewers. Any product that may be evaluated in this article, or claim that may be made by its manufacturer, is not guaranteed or endorsed by the publisher.

## Supplementary Material

Supplementary Figure 1 The growth curves of genetically modified strain and WT strain. The E1, E2 and E3 were clone lines for the expression of hDHFR-eGFP, and T1, T2 and T3 were clone lines for TPX-1 KO parasites.

## Abbreviations

ef-1α, elongation factor-1α; *TPX-1*, *thioredoxin peroxidase-1*; IC50, half-maximum inhibition concentration; PPE, percent parasitized erythrocytes.

## References

[B1] Abraham A. Brasov I. Thekkiniath J. Kilian N. Lawres L. Gao R.. (2018). Establishment of a Continuous *In Vitro* Culture of Babesia Duncani in Human Erythrocytes Reveals Unusually High Tolerance to Recommended Therapies. J. Biol. Chem. 293, 19974–19981. doi: 10.1074/jbc.AC118.005771 30463941 PMC6311517

[B2] Adamson R. Lyons K. Sharrard M. Kinnaird J. Swan D. Graham S.. (2001). Transient Transfection of Theileria Annulata. Mol. Biochem. Parasitol. 114, 53–61. doi: 10.1016/S0166-6851(01)00238-9 11356513

[B3] Alzan H. F. Cooke B. M. Suarez C. E. (2019). Transgenic Babesia Bovis Lacking 6-Cys Sexual-Stage Genes as the Foundation for Non-Transmissible Live Vaccines Against Bovine Babesiosis. Ticks Tick-Borne Dis. 10, 722–728. doi: 10.1016/j.ttbdis.2019.01.006 30711475

[B4] Asada M. Tanaka M. Goto Y. Yokoyama N. Inoue N. Kawazu S. (2012). Stable Expression of Green Fluorescent Protein and Targeted Disruption of Thioredoxin Peroxidase-1 Gene in Babesia Bovis With the WR99210/dhfr Selection System. Mol. Biochem. Parasitol. 181, 162–170. doi: 10.1016/j.molbiopara.2011.11.001 22108434

[B5] Asada M. Yahata K. Hakimi H. Yokoyama N. Igarashi I. Kaneko O.. (2015). Transfection of Babesia Bovis by Double Selection With WR99210 and Blasticidin-S and Its Application for Functional Analysis of Thioredoxin Peroxidase-1. PloS One 10, e0125993. doi: 10.1371/journal.pone.0125993 25962142 PMC4427477

[B6] Braga W. Venasco J. Willard L. Moro M. H. (2006). Ultrastructure of Babesia WA1 (Apicomplexa: Piroplasma) During Infection of Erythrocytes in a Hamster Model. J. Parasitol. 92, 1104–1107. doi: 10.1645/GE-712R.1 17152960

[B7] Conrad P. A. Kjemtrup A. M. Carreno R. A. Thomford J. Wainwright K. Eberhard M.. (2006). Description of Babesia Duncani N.Sp. (Apicomplexa: Babesiidae) From Humans and Its Differentiation From Other Piroplasms. Int. J. Parasitol. 36, 779–789. doi: 10.1016/j.ijpara.2006.03.008 16725142

[B8] Dao A. H. Eberhard M. L. (1996). Pathology of Acute Fatal Babesiosis in Hamsters Experimentally Infected With the WA-1 Strain of Babesia. Lab. Invest. J. Tech. Methods Pathol. 74, 853–859. 8642781

[B9] De Goeyse I. Jansen F. Madder M. Hayashida K. Berkvens D. Dobbelaere D.. (2015). Transfection of Live, Tick Derived Sporozoites of the Protozoan Apicomplexan Parasite Theileria Parva. Vet. Parasitol. 208, 238–241. doi: 10.1016/j.vetpar.2015.01.013 25660425

[B10] de Koning-Ward T. F. Fidock D. A. Thathy V. Menard R. van Spaendonk R. M. Waters A. P.. (2000). The Selectable Marker Human Dihydrofolate Reductase Enables Sequential Genetic Manipulation of the Plasmodium Berghei Genome. Mol. Biochem. Parasitol. 106, 199–212. doi: 10.1016/S0166-6851(99)00189-9 10699250

[B11] Gallego-Lopez G. M. Lau A. O. T. O'Connor R. M. Ueti M. W. Cooke B. M. Laughery J. M.. (2019). Up-Regulated Expression of Spherical Body Protein 2 Truncated Copy 11 in Babesia Bovis Is Associated With Reduced Cytoadhesion to Vascular Endothelial Cells. Int. J. Parasitol. 49, 127–137. doi: 10.1016/j.ijpara.2018.05.015 30367864

[B12] Hakimi H. Yamagishi J. Kegawa Y. Kaneko O. Kawazu S. Asada M. (2016). Establishment of Transient and Stable Transfection Systems for Babesia Ovata. Parasites Vectors 9, 171. doi: 10.1186/s13071-016-1439-z 27008652 PMC4806448

[B13] Hemmer R. M. Wozniak E. J. Lowenstine L. J. Plopper C. G. Wong V. Conrad P. A. (1999). Endothelial Cell Changes Are Associated With Pulmonary Edema and Respiratory Distress in Mice Infected With the WA1 Human Babesia Parasite. J. Parasitol. 85, 479–489. doi: 10.2307/3285783 10386441

[B14] Jaijyan D. K. Govindasamy K. Singh J. Bhattacharya S. Singh A. P. (2020). Establishment of a Stable Transfection Method in Babesia Microti and Identification of a Novel Bidirectional Promoter of Babesia Microti. Sci. Rep. 10, 15614. doi: 10.1038/s41598-020-72489-3 32973208 PMC7515924

[B15] Kim T. Y. Kim S. Y. Kim T. K. Lee H. I. Cho S. H. Lee W. G.. (2021). Molecular Evidence of Zoonotic Babesia Species, Other Than B. Microti, in Ixodid Ticks Collected From Small Mammals in the Republic of Korea. Vet Med. Sci. 7 (6), 2427–2423. doi: 10.1002/vms3.581 34492740 PMC8604135

[B16] Kjemtrup A. M. Conrad P. A. (2000). Human Babesiosis: An Emerging Tick-Borne Disease. Int. J. Parasitol. 30, 1323–1337. doi: 10.1016/S0020-7519(00)00137-5 11113258

[B17] Klevens R. M. Cumming M. A. Caten E. Stramer S. L. Townsend R. L. Tonnetti L.. (2018). Transfusion-Transmitted Babesiosis: One State's Experience. Transfusion 58, 2611–2616. doi: 10.1111/trf.14943 30260481

[B18] Liu M. Adjou Moumouni P. F. Asada M. Hakimi H. Masatani T. Vudriko P.. (2018). Establishment of a Stable Transfection System for Genetic Manipulation of Babesia Gibsoni. Parasites Vectors 11, 260. doi: 10.1186/s13071-018-2853-1 29685172 PMC5914073

[B19] McCormack K. A. Alhaboubi A. Pollard D. A. Fuller L. Holman P. J. (2019). *In Vitro* Cultivation of Babesia Duncani (Apicomplexa: Babesiidae), a Zoonotic Hemoprotozoan, Using Infected Blood From Syrian Hamsters (Mesocricetus Auratus). Parasitol. Res. 118, 2409–2417. doi: 10.1007/s00436-019-06372-0 31197543

[B20] Mira-Martínez S. Rovira-Graells N. Crowley V. M. Altenhofen L. M. Llinás M. Cortés A. (2013). Epigenetic Switches in Clag3 Genes Mediate Blasticidin S Resistance in Malaria Parasites. Cell. Microbiol. 15, 1913–1923. doi: 10.1111/cmi.12162 23819786 PMC4621952

[B21] Moro M. H. David C. S. Magera J. M. Wettstein P. J. Barthold S. W. Persing D. H. (1998). Differential Effects of Infection With a Babesia-Like Piroplasm, WA1, in Inbred Mice. Infect. Immun. 66, 492–498. doi: 10.1128/IAI.66.2.492-498.1998 9453601 PMC107933

[B22] O'Connor K. E. Kjemtrup A. M. Conrad P. A. Swei A. (2018). An Improved PCR Protocol For Detection of Babesia duncanI In Wildlife and Vector Samples. J. Parasitol. 104, 429–432. doi: 10.1645/17-155 29659338

[B23] Quick R. E. Herwaldt B. L. Thomford J. W. Garnett M. E. Eberhard M. L. Wilson M.. (1993). Babesiosis in Washington State: A New Species of Babesia? Ann. Internal Med. 119, 284–290. doi: 10.7326/0003-4819-119-4-199308150-00006 8328736

[B24] Sahi J. Shord S. S. Lindley C. Ferguson S. LeCluyse E. L. (2009). Regulation of Cytochrome P450 2C9 Expression in Primary Cultures of Human Hepatocytes. J. Biochem. Mol. Toxicol. 23, 43–58. doi: 10.1002/jbt.20264 19202563

[B25] Scott J. D. Scott C. M. (2018). Human Babesiosis Caused by Babesia Duncani Has Widespread Distribution Across Canada. Healthcare (Basel Switzerland) 6(2), 49. doi: 10.3390/healthcare6020049 PMC6023460 29772759

[B26] Suarez C. E. Bishop R. P. Alzan H. F. Poole W. A. Cooke B. M. (2017). Advances in the Application of Genetic Manipulation Methods to Apicomplexan Parasites. Int. J. Parasitol. 47, 701–710. doi: 10.1016/j.ijpara.2017.08.002 28893636

[B27] Suarez C. E. McElwain T. F. (2008). Transient Transfection of Purified Babesia Bovis Merozoites. Exp. Parasitol. 118, 498–504. doi: 10.1016/j.exppara.2007.10.013 18076879

[B28] Suarez C. E. McElwain T. F. (2009). Stable Expression of a GFP-BSD Fusion Protein in Babesia Bovis Merozoites. Int. J. Parasitol. 39, 289–297. doi: 10.1016/j.ijpara.2008.08.006 18831975

[B29] Suarez C. E. McElwain T. F. (2010). Transfection Systems for Babesia Bovis: A Review of Methods for the Transient and Stable Expression of Exogenous Genes. Vet. Parasitol. 167, 205–215. doi: 10.1016/j.vetpar.2009.09.022 19819628

[B30] Suarez C. E. Noh S. (2011). Emerging Perspectives in the Research of Bovine Babesiosis and Anaplasmosis. Vet. Parasitol. 180, 109–125. doi: 10.1016/j.vetpar.2011.05.032 21684084

[B31] Suarez C. E. Norimine J. Lacy P. McElwain T. F. (2006). Characterization and Gene Expression of Babesia Bovis Elongation Factor-1alpha. Int. J. Parasitol. 36, 965–973. doi: 10.1016/j.ijpara.2006.02.022 16677650

[B32] Swei A. O'Connor K. E. Couper L. I. Thekkiniath J. Conrad P. A. Padgett K. A.. (2019). Evidence for Transmission of the Zoonotic Apicomplexan Parasite Babesia Duncani by the Tick Dermacentor Albipictus. Int. J. Parasitol. 49, 95–103. doi: 10.1016/j.ijpara.2018.07.002 30367862 PMC10016146

[B33] Vannier E. G. Diuk-Wasser M. A. Ben Mamoun C. Krause P. J. (2015). Babesiosis. Infect. Dis. Clin North Am 29, 357–370. doi: 10.1016/j.idc.2015.02.008 25999229 PMC4458703

[B34] Vannier E. Krause P. J. (2012). Human Babesiosis. N Engl. J. Med. 366, 2397–2407. doi: 10.1056/NEJMra1202018 22716978

[B35] Villatoro T. Karp J. K. (2019). Transfusion-Transmitted Babesiosis. Arch. Pathol. Lab. Med. 143, 130–134. doi: 10.5858/arpa.2017-0250-RS 30376376

[B36] Virji A. Z. Thekkiniath J. Ma W. Lawres L. Knight J. Swei A.. (2019). Insights Into the Evolution and Drug Susceptibility of Babesia Duncani From the Sequence of its Mitochondrial and Apicoplast Genomes. Int. J. Parasitol. 49, 105–113. doi: 10.1016/j.ijpara.2018.05.008 30176236 PMC6395566

[B37] Wang J. Wang X. Guan G. Yang J. Liu J. Liu A.. (2021). Stable Transfection System for Babesia Sp. Xinjiang. Parasites Vectors 14, 463. doi: 10.1186/s13071-021-04940-x 34503543 PMC8428105

[B38] Wozniak E. J. Lowenstine L. J. Hemmer R. Robinson T. Conrad P. A. (1996). Comparative Pathogenesis of Human WA1 and Babesia Microti Isolates in a Syrian Hamster Model. Lab. Anim. Sci. 46, 507–515. 8905583

[B39] Yabsley M. J. Shock B. C. (2013). Natural History of Zoonotic Babesia: Role of Wildlife Reservoirs. Int. J. Parasitol. Parasites Wildlife 2, 18–31. doi: 10.1016/j.ijppaw.2012.11.003 PMC3862492 24533312

