# Supplementary figures and images for: Establishment of a Transient and Stable Transfection System for Babesia duncani Using a Homologous Recombination Strategy
Source: Front Cell Infect Microbiol. 2022 Apr 6;12:844498. doi: 10.3389/fcimb.2022.844498 (PMC9019647; doi:10.3389/fcimb.2022.844498)

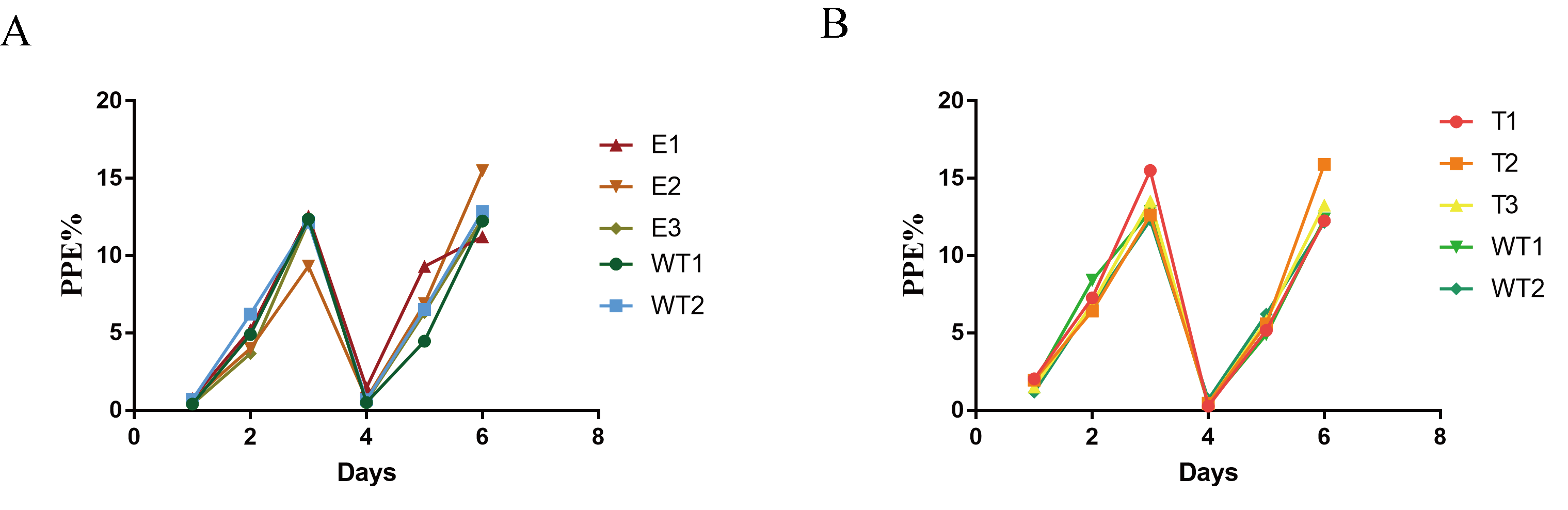

Supplement: Supplementary Figure 1 — The growth curves of genetically modified strain and WT strain. The E1, E2 and E3 were clone lines for the expression of hDHFR-eGFP, and T1, T2 and T3 were clone lines for TPX-1 KO parasites. [file Image_1.tif]
